# Robotic Ultrasound Scanning With Real-Time Image-Based Force Adjustment: Quick Response for Enabling Physical Distancing During the COVID-19 Pandemic

**DOI:** 10.3389/frobt.2021.645424

**Published:** 2021-03-22

**Authors:** Mojtaba Akbari, Jay Carriere, Tyler Meyer, Ron Sloboda, Siraj Husain, Nawaid Usmani, Mahdi Tavakoli

**Affiliations:** ^1^Telerobotic and Biorobotic System Group, Electrical and Computer Engineering, University of Alberta, Edmonton, AB, Canada; ^2^Division of Radiation Oncology, Tom Baker Cancer Centre, Calgary, AB, Canada; ^3^Department of Oncology, Cross Cancer Institute, Edmonton, AB, Canada

**Keywords:** medical image quality assessment, medical robotic, ultrasound scanning, artificial intelligence, robotics for COVID-19

## Abstract

During an ultrasound (US) scan, the sonographer is in close contact with the patient, which puts them at risk of COVID-19 transmission. In this paper, we propose a robot-assisted system that automatically scans tissue, increasing sonographer/patient distance and decreasing contact duration between them. This method is developed as a quick response to the COVID-19 pandemic. It considers the preferences of the sonographers in terms of how US scanning is done and can be trained quickly for different applications. Our proposed system automatically scans the tissue using a dexterous robot arm that holds US probe. The system assesses the quality of the acquired US images in real-time. This US image feedback will be used to automatically adjust the US probe contact force based on the quality of the image frame. The quality assessment algorithm is based on three US image features: correlation, compression and noise characteristics. These US image features are input to the SVM classifier, and the robot arm will adjust the US scanning force based on the SVM output. The proposed system enables the sonographer to maintain a distance from the patient because the sonographer does not have to be holding the probe and pressing against the patient's body for any prolonged time. The SVM was trained using bovine and porcine biological tissue, the system was then tested experimentally on plastisol phantom tissue. The result of the experiments shows us that our proposed quality assessment algorithm successfully maintains US image quality and is fast enough for use in a robotic control loop.

## 1. Introduction

Ultrasound (US) image acquisition is a popular medical imaging method because it does not involve radiation (like x-ray or CT do), is generally regarded as safe, has a low cost compared to other medical imaging methods and is widely available. For a healthcare system that is struggling with COVID-19, US scanning is a way for COVID-19 diagnosis (Buda et al., 2020; McDermott et al., 2020), especially in developing countries where access to the lab kit is very limited. But there are some factors regarding the US scanning procedure during COVID-19 pandemic that need to be addressed. The first factor is the close contact between sonographers and patients; it is very important to minimize contact between sonographers and patients during the COVID-19 pandemic. It has been proven that close person/person contact is the main way for the transmission of the virus (Jarvis et al., 2020; Jin et al., 2020; Morawska and Milton, 2020; Zu et al., 2020). The second factor is related to COVID-19 patients with underlying conditions such as heart conditions. These patients are at heightened risk, and some of these underlying conditions need US imaging, like echocardiography. The third factor is that US imaging can also be quite time-consuming. Most US scans last between 15 and 45 min (NHL, 2018). For example, echocardiography takes almost 20 min (Ebadollahi et al., 2001). Because of this, we need a system that helps a sonographer to scan tissue and decreases the contact duration (i.e., allows for greater distancing) between sonographers and patients. This paper proposes a quick, low-cost, and deployable solution for the problem mentioned above as a consequence of the COVID-19 pandemic. Robots can be very useful for solving this problem. The part of the scanning that requires experience and knowledge of the sonographer can be done the normal way, and the parts that put the sonographer at an increased risk of contacting the virus can be delegated to the robotic system just like the way x-ray systems work. Using robots during the COVID-19 pandemic can significantly decrease the risk of virus transmission (Tavakoli et al., 2020) particularly because the proposed robotic system can be sanitized between each US scanning procedure.

The assessment of image quality is essential in developing robotic US scanning. Image quality assessment has been a challenging topic in medical image processing, and different methods have been proposed in the literature. There are three different categories of image quality assessment algorithm based on the availability of reference images or other supplementary information. The first category is called full-reference image quality assessment. A reference image (high-quality image) is available in this category, and the quality assessment metric is implemented by comparing a given image to the reference image. The second category is called semi-reference image quality assessment, in which the algorithm has access only to some information about the reference image, such as important features in the image. For instance, Chen et al. (2020) uses the visual features (statistical features from contourlet transform) that are critical for both human perception and object recognition for sonar image quality assessment, but the reference image is not available. Semi-reference methods are more challenging than full-reference algorithms, and how to utilize the additional information is an important question for these algorithms. The final category is called no-reference image quality assessment, in which the algorithm does not have access to the reference image or any additional information related to it. This category is the most challenging but is very important and useful for medical image quality assessment (Chow and Paramesran, 2016). Being as typically we do not have access to quality reference images, the crucial part of no-reference image quality assessment is developing the quality metrics. Quality metrics should be based on features that are present in either high-quality or low-quality images. The extracted features need to be combined to build a quality metric that creates an image quality score.

**Figure 1:**
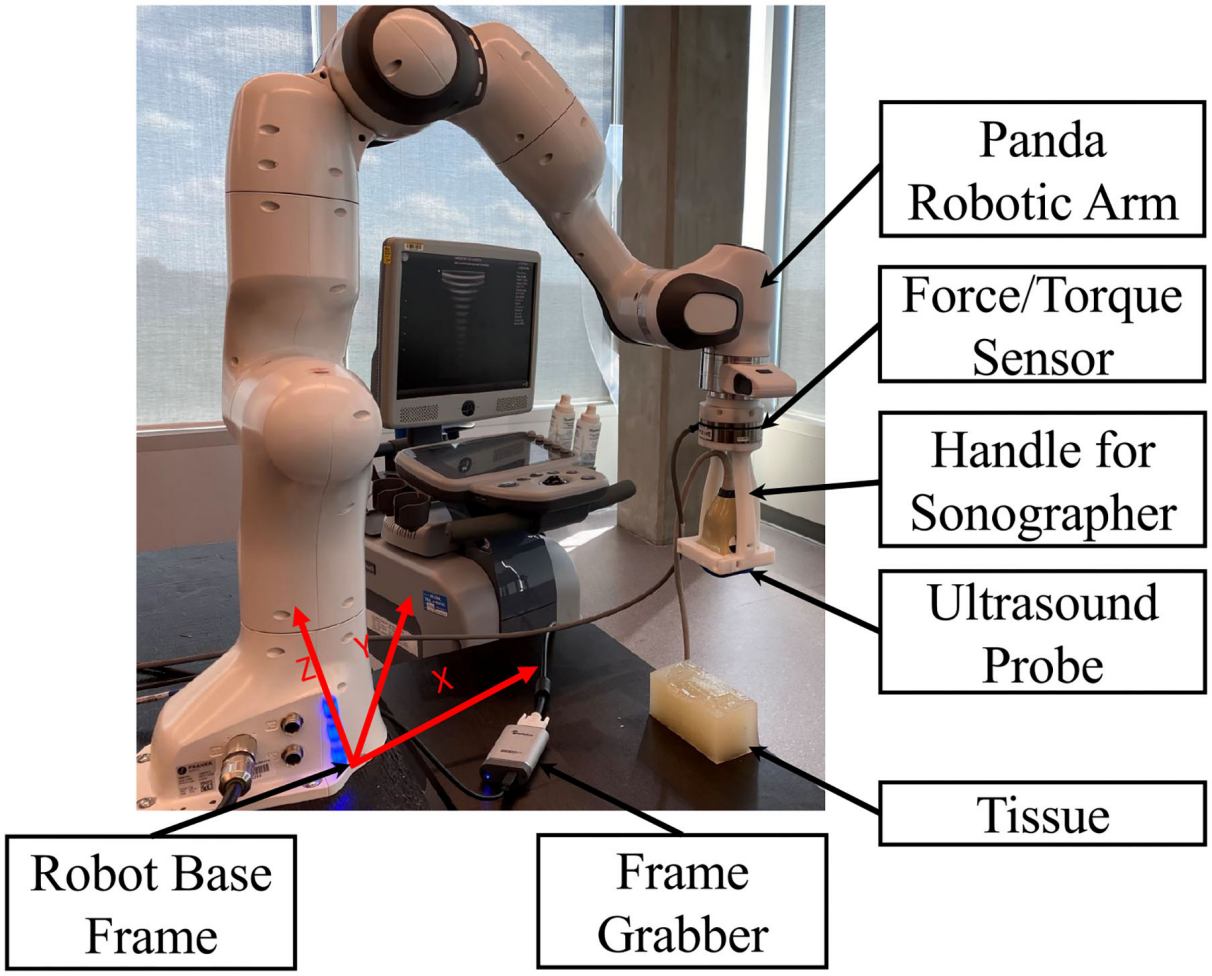
US scanning assistant including Panda robot arm, US probe, handle for sonographer, tissue phantom, frame grabber, and robot base frame.

The problem with US images processing is the inherent noise in the images, making it difficult for a physician to interpret them. This makes US image quality assessment a very complicated task. In this paper, we propose a method for assessing the US image's quality when a robotic arm is holding the US probe. We will incorporate the algorithm in the robot control loop for automatic scanning of tissue. An admittance-based controller will be used for the robot and automatically control the probe's scanning force applied to the tissue. The admittance controller produces a desired position using a predefined relationship between the position and measured force (Zeng and Hemami, 1997; Fong and Tavakoli, 2018). The US scanning assistant is shown in Figure 1. The sonographer uses a handle to position the robot by incorporating a robot's built-in admittance control, and the robot adjusts the US scanning force applied to the tissue by analyzing the quality of the acquired image. This system reduces contact time and mitigates the risk of virus transmission between the sonographer and the patient. Being as the system scans the tissue based on image quality assessment feedback, the sonographer does not need to be next to the patient for the whole duration of the scanning.

The outline of the paper is as follows. In section 2, we will give a brief review of previous medical image quality assessment algorithms, robot-assisted sonography and robotic admittance control applications. We will address the contributions of this paper in section 2.4. We develop our proposed image quality assessment algorithm in section 3 by giving details of the algorithm and discussing the specific image features it uses. In section 4, we will give the details of the robotic admittance controller used in the system to adjust the US scanning force applied to the tissue. The experimental setup and the experimental results are presented in section 5. We will conclude our method and its advantages in section 6.

## 2. Prior Work

In this section, we will talk about previous work that has been done in medical image quality assessment, robot-assisted sonography, and robotic admittance control. We will talk about our contribution and novelty in the last paragraph of this section.

### 2.1. Medical Image Quality Assessment

Medical image quality assessment is a broad topic across multiple imaging modalities, with each imaging modality having its features and characteristics that need to be considered. A review of different medical image quality assessment algorithms and their corresponding imaging modalities can be found in Chow and Paramesran (2016). The most crucial problem in medical image quality assessment is the unavailability of reference data, and most methods are based on no-reference image quality assessment algorithms. We can classify no-reference medical image quality assessment methods into model-based and image-based methods. The algorithm is based on modeling both images and noise in a model-based image quality assessment algorithm, such as the method proposed in Zemp et al. (2003). On the other hand, in image-based quality assessment algorithms, metrics are present to assess the image's quality.

In US image quality assessment, different methods have been proposed for modeling image and noise. In Zemp et al. (2003), the author uses Noise-Equivalent Quanta (NEQ) that models noise based on US machine parameters and tissue physical property information; an improved version of the signal-to-noise ratio. Structural Similarity Index Measure (SSIM) is a very famous image quality assessment metric and has been used in many different applications. The method proposed in Renieblas et al. (2017) uses SSIM as the main quality assessment criteria and incorporates specific image features like preserved edges, structural similarity, and textures in the image.

Image-based quality assessment methods propose criteria that formalize critical features for quality assessment. The method proposed in Hemmsen et al. (2010) uses data management and data acquisition techniques to formalize the quality assessment metrics for US images. The authors of Abdel-Hamid et al. (2016) use five important features of transformed images for building a quality assessment metric. These five features are sharpness, illumination, homogeneity, field definition, and content. The method proposed in Abdel-Hamid et al. (2016) uses the wavelet transform and extracts the five image features from the transformed image, and combining them to create a formula for image quality assessment of human retina images.

As one modality of medical imaging, US poses many challenges in terms of image quality assessment. These challenges come primarily from the noisy nature of the US images. US image's quality is usually defined as an ability to see some tissue features or organs in the image. The method proposed in Zhang et al. (2017) developed a method of segmenting the fetus in an US image, using a texton method on the image. The texton method performs segmentation and feature extraction, and a random forest classifier assesses the quality of the image based on the extracted features. Schwaab et al. (2016) proposes the extraction of three features from breast US images and uses a random forest for classification of those. These features are the nipple position, the shadow caused by the nipple, and the breast contours' shape. Schinaia et al. (2017) used a method similar to Schwaab et al. (2016), but incorporated 14 features and a correlation matrix for quality assessment. Deep Convolutional Neural Networks (CNN) have also proven to perform well for complicated tasks like this. Wu et al. (2017) uses two deep convolutional neural networks called C-CNN and L-CNN for quality assessment. L-CNN finds an ROI (Region Of Interest) in the image, and C-CNN evaluates the quality of the image based on the extracted ROI. The output of C-CNN is the binary label segmentation of the US image. The method proposed in Chatelain et al. (2015) and Welleweerd et al. (2020) use confidence map, which was proposed in Karamalis et al. (2012) for orienting and moving the US probe during scanning of the tissue. Confidence map methods are based on US signal propagation model inside of the tissue and the outcome is a map that can be used for feature extraction. The extracted features are the inputs to the controller and the output is the control signals for controlling the probe's orientation and position.

### 2.2. Robot-Assisted Sonography

Robots can be very helpful to a sonographer during US scanning. Many methods have been proposed to facilitate the process of sonography using robots. Najafi and Sepehri (2011) developed a robotic wrist to perform US imaging on a patient at remote sites. This system has four degrees of freedom and has been used for US imaging of the liver and kidney. The device developed in AbbasiMoshaii and Najafi (2019) is placed on the patient's body by an operator, and US expert controls the device's motion to obtain US image. The paper focuses on the robotic mechanism that performs US imaging. The mechanism keeps the US probe in contact with the patient's body and facilitates the sonographer's US scanning procedure. Fang et al. (2017) proposes a cooperatively robotic US system to reduce the force sonographers apply. This system consists of a six-axis robotic arm that holds and actuates the US probe. A dual force sensor setup enables cooperative control and adaptive force assistance using admittance force control. Antico et al. (2019) prepared a good review of different methods proposed in robot-assisted US intervention, and Moshaii and Najafi (2014) is a good review of the mechanical details of robot-assisted US scanning.

Tele-sonography is developed for scanning the tissue using remote robot. Sharifi et al. (2017) developed an impedance-controlled teleoperation system for robot-assisted tele-echography of moving organs such as heart, chest, and breast compensating for their natural motions. This system proposes two impedance models for master and slave robots. The slave robot follows the master robot's trajectory but complies with the oscillatory interaction force of moving organs, and the sonographer receives feedback from the slave robot. Sartori et al. (2019) proposes a solution for energy consumption in tele-echography on the master site based on properly scaling the energy exchanged between the master and the slave site. There are many challenges in designing tele-sonography system. The most important one is the high cost of the system and haptic feedback required in the master site. Using haptic feedback causes time delay in the system that may result discrepancy between sonographer and US probe during scanning. Our proposed method can be used as a local controller in the slave site to overcome this problem.

### 2.3. Robot Admittance Control

Admittance controller uses a predefined relationship between force and position. Authors in Carriere et al. (2019) use admittance control to ensure compliance in a co-manipulated US scanning system controlling the force applied to the tissue and reducing exerted force from the sonographer. The method proposed in Piwowarczyk et al. (2020) uses an admittance controller to scale the force applied by the user on the robot in relation to force applied to the environment. The stability of admittance-controlled robots and their ability to cope with different environmental forces have been investigated in Ferraguti et al. (2019). Admittance control was used in Li et al. (2018) for an exoskeleton robot to create a reference trajectory based on measured force. Dimeas and Aspragathos (2016) analyzes the stability of admittance control by detecting unstable behaviors and stabilizing the robot using an adaptive online method to tune the admittance control gains. The stabilization of the robot is based on monitoring high-frequency oscillations in the force signals. This idea was also used in Landi et al. (2017) for stabilizing the admittance control when interacting with humans. The idea of incorporating neural networks and admittance control for robot trajectory tracking is developed in Yang et al. (2018), in which the trajectory tracking is guaranteed by using a neural network while admittance control regulates torques to follow the desired trajectory. Authors in Keemink et al. (2018) prepared a very good review of different applications of admittance control in robotics.

### 2.4. Contributions of This Paper

As we mentioned in section 2.3, different methods and applications have been proposed for medical image quality assessment and robotic admittance control but all of them do not consider image feedback in admittance controllers. The idea of combining image feedback and admittance controller in the US scanning procedure is the first novelty of this paper. We also allow for collaboration between humans and the robot to keep the sonographer in the loop during the US scanning procedure. The proposed method uses a real-time image quality assessment algorithm to inform the robotic system. The real-time nature of the proposed image quality assessment algorithm makes it suitable for the clinician in the loop robot-assisted medical applications. The combination of admittance control and online image quality assessment algorithm in the robotic arm ensures social distancing during the COVID-19 pandemic and has not been explored before in the literature.

The second novelty of this paper is to propose a very quick, low-cost, and deployable solution for the COVID-19 pandemic that can be trained based on the preferences of the sonographers in terms of how US scanning is done in different applications. The training phase requires nothing more than the commodity hardware (e.g., a personal computer). This is a very important advantage of the proposed system over the method mentioned in section 2.1. The proposed method has the ability to consider the preferences of the sonographers for different applications by incorporating it in the training phase. The sonographer can manually classify the training set and the system will tune the parameters for the sonographer's preferences. To the best of our knowledge, this ability has not been investigated in the previous methods.

The third novelty of the proposed method is the ability to be used in unilateral tele-sonography as a controller on the patient side. In a tele-sonography modality, the sonographer moves the robot to the desired position using a master robot. The sonographer needs to feel the contact force between the tissue and the probe during scanning. The system should have a haptic interface on the master site to enable this feature for the sonographer. Using a haptic interface could cause a time delay in the system during scanning as discussed in Najafi and Sepehri (2011), Sharifi et al. (2017), Moshaii and Najafi (2014), and AbbasiMoshaii and Najafi (2019). The low-cost and better solution is using a unilateral tele-sonography system with a local controller on the patient site that adjusts the force applied to the tissue during scanning based on acquired image's quality. Our proposed method can be incorporated as a local controller in the slave site to adjust the force applied to the tissue based on the preferences of the sonographers. This feature will remove the essence of having haptic feedback in the tele-sonography system and will decrease the cost of the system.

## 3. Image Quality Assessment Algorithm

As previously mentioned, US images are usually very noisy, and therefore, the tissue is not very clear in the images. This problem makes the automated assessment of US images complicated. A US image quality assessment algorithm should distinguish between different features in an image and decide on image quality based on the acquired features. For our proposed image quality assessment method, we will use a Support Vector Machine (SVM) classifier, which is compatible with small training sets and has proven to have a good ability to solve complicated problems, especially in medical applications.

### 3.1. Image Quality Assessment Metrics

We propose three distinct features for estimating the quality of the image. The first feature is based on the contact between the probe and the tissue. The second feature computes the level of compression caused by the US scanning force applied to the tissue. The third feature is an estimation of the noise level in the image. The noise level is estimated based on the statistical features of the noise in the US image. We will discuss each of the features in-depth in the following sub-sections.

#### 3.1.1. Correlation

**Figure 2:**
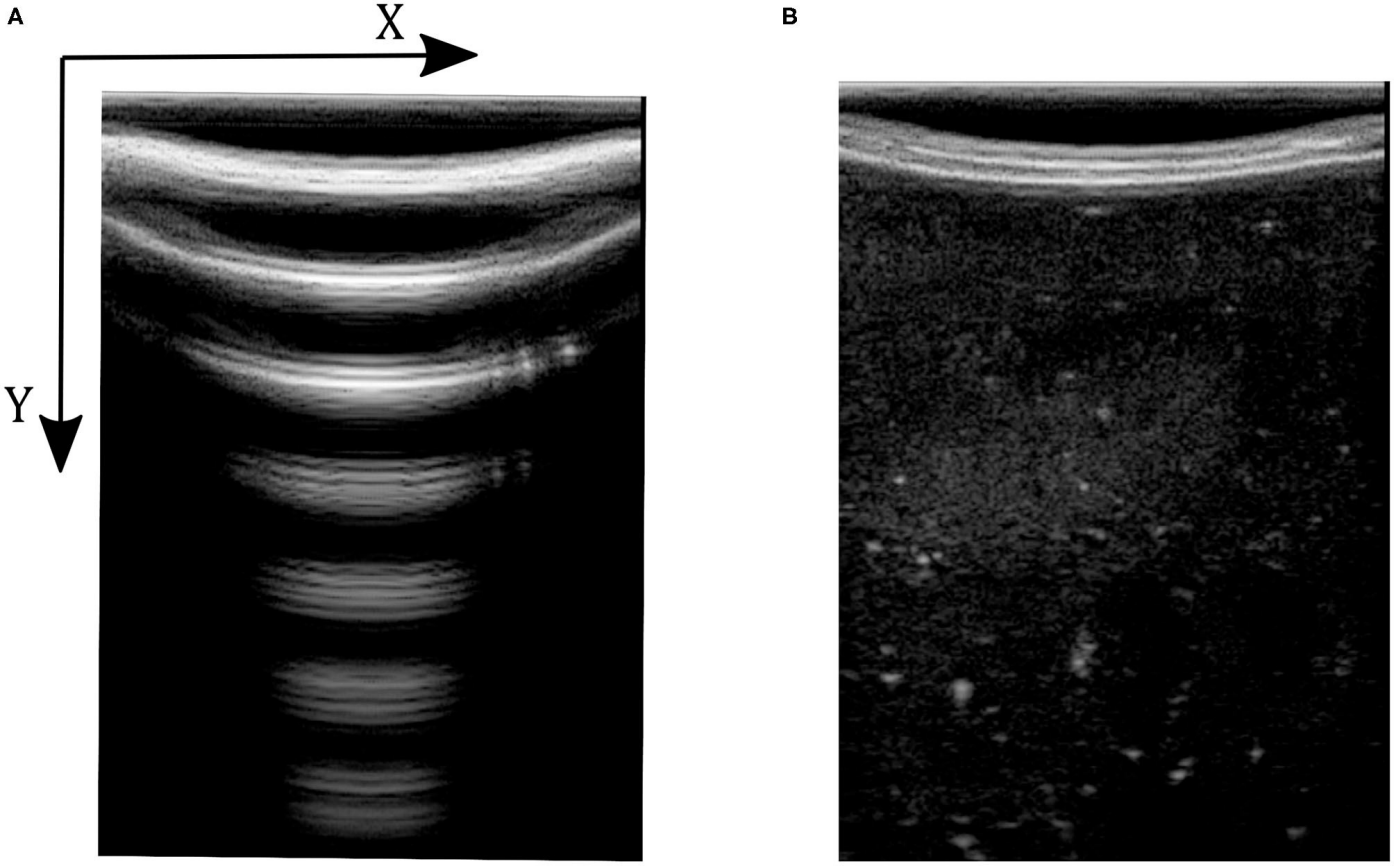
Ultrasound images with and without contact between tissue and probe. **(A)** Ultrasound image with no contact. **(B)** Ultrasound image with sufficient contact.

We use image correlation for modeling the contact between the tissue and probe. When there is no contact (or proper contact) between the probe and tissue, the US image will only consist of patterns of arcs; see Figure 2A. When we have sufficient contact, however, actual tissue will be visible in the image. In Figure 2A, the image captured by the US machine was defined as no-contact image *I*_*nc*_ in the sense that probe is not contacting the tissue when the image is captured. We define the contact feature as the correlation of no-contact image *I*_*nc*_ with an image captured by the US machine *I*_*k*_ in every time step *k* of the experiment. The contact feature *c*_*k*_ gives us a good estimation of the sufficiency of contact and *c*_*k*_ ∈ [0, 1]. The mathematical details of how the correlation between the images is calculated and how contact between the probe and the tissue is defined are as follows:

(1) $$\begin{array}{r} \text{corr}\left({\text{I}}_{\text{k}},{\text{I}}_{\text{nc}}\right)\\ =\frac{\sum_{p_{x}=1}^{M}\sum_{p_{y}=1}^{N}\left(I_{k}\left(p_{x},p_{y}\right)-\bar{I_{k}}\right)\left(I_{nc}\left(p_{x},p_{y}\right)-\bar{I_{nc}}\right)}{\sqrt{\left(\sum_{p_{x}=1}^{M}\sum_{p_{y}=1}^{N}{\left(I_{k}\left(p_{x},p_{y}\right)-\bar{I_{k}}\right)}^{2}\right)\left(\sum_{p_{x}=1}^{M}\sum_{p_{y}=1}^{N}{\left(I_{nc}\left(p_{x},p_{y}\right)-\bar{I_{nc}}\right)}^{2}\right)}} \end{array}$$

(2) $$\begin{array}{r} c_{k}=\left\{ \begin{array}{cc} 1, & \text{ifcorr}\left({\text{I}}_{\text{k}},{\text{I}}_{\text{nc}}\right)\geq \;t_{corr}\\ 0, & \text{ifcorr}\left({\text{I}}_{\text{k}},{\text{I}}_{\text{nc}}\right)<\;t_{corr} \end{array}\right. \end{array}$$

Here, the contact feature *c*_*k*_ is the value of the correlation between the two images. (*p*_*x*_, *p*_*y*_) is the location of pixels in the image frame, and *M* and *N* are the height and width of input images, respectively. $\bar{I_{k}}$ and $\bar{I_{nc}}$ are the average of the pixels' intensities in the acquired image and the image with no contact with the tissue, respectively, and *t*_*corr*_ is the threshold for determining the contact level. Figure 2 shows two images, in which Figure 2A was captured when there is not enough contact between the tissue and the probe, and Figure 2B was conducted with sufficient contact. The x-y axis in the image frame is shown in Figure 2A and it is the same for all images in this paper.

#### 3.1.2. Compression

The level of compression is a very important feature in US image acquisition. When the robot applies force to the tissue, it causes deformation. More force causes greater distortion/deformation. This causes pain for the patient, and may lead to wrong clinical diagnosis (Fang et al., 2017). The proposed compression feature is the difference between the maximum and minimum index of the pixels brighter than the threshold *t*_*comp*_, relative to the image's size in the vertical direction. The mathematical expression for calculating the image compression feature is as follows:

(3) $$\begin{array}{r} \begin{array}{c} U=\max\left(p_{y}\right),\;\text{where }I_{k}\left(p_{y},\forall p_{x}\in I_{k}\right)\geq t_{comp}\\ L=\min\left(p_{y}\right),\;\text{where }I_{k}\left(p_{y},\forall p_{x}\in I_{k}\right)\geq t_{comp}\\ f_{c}=\frac{U-L}{M} \end{array} \end{array}$$

**Figure 3:**
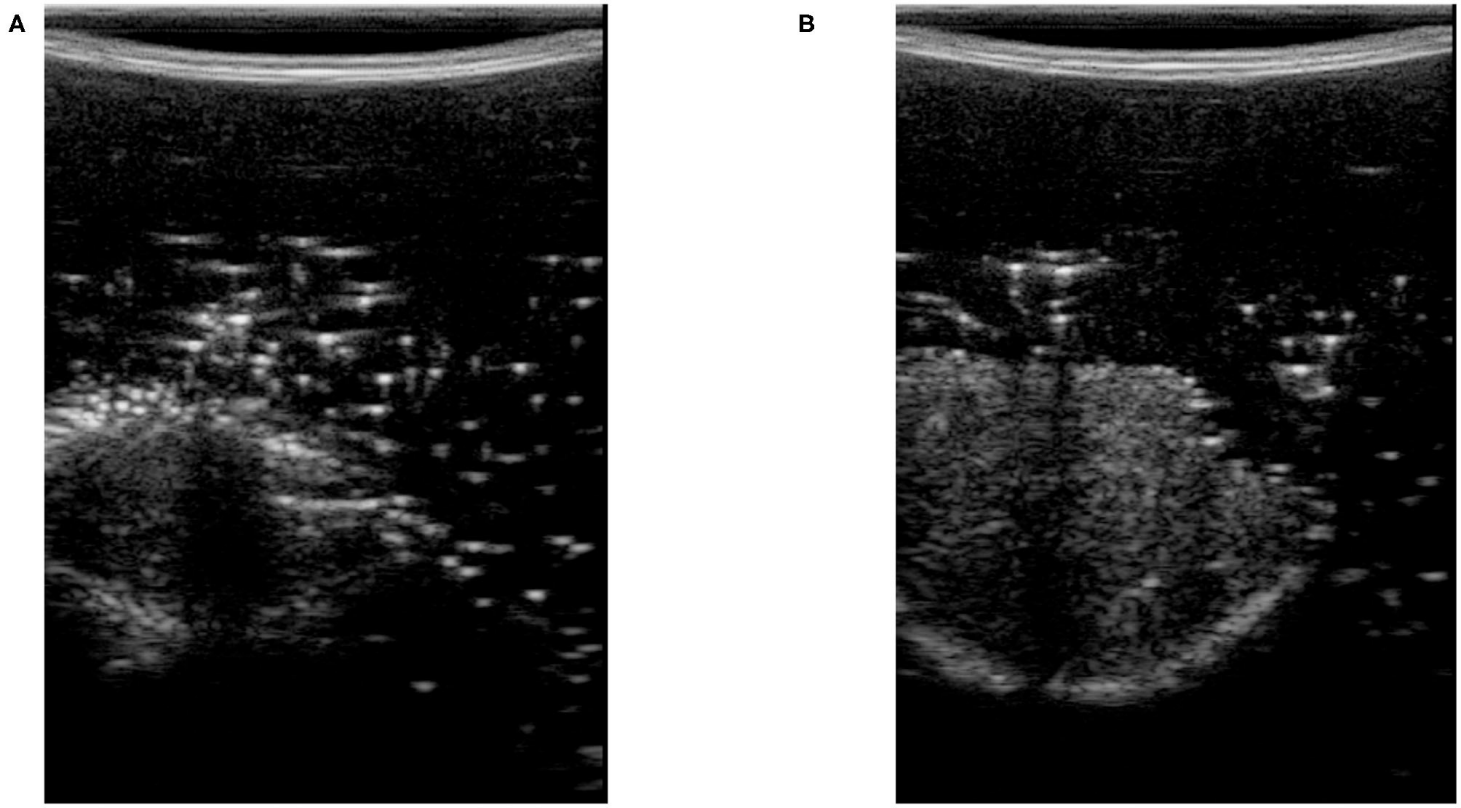
Ultrasound images with high and low level of compression. **(A)** Ultrasound image with high tissue compression. **(B)** Ultrasound image with low tissue compression.

**Figure 4:**
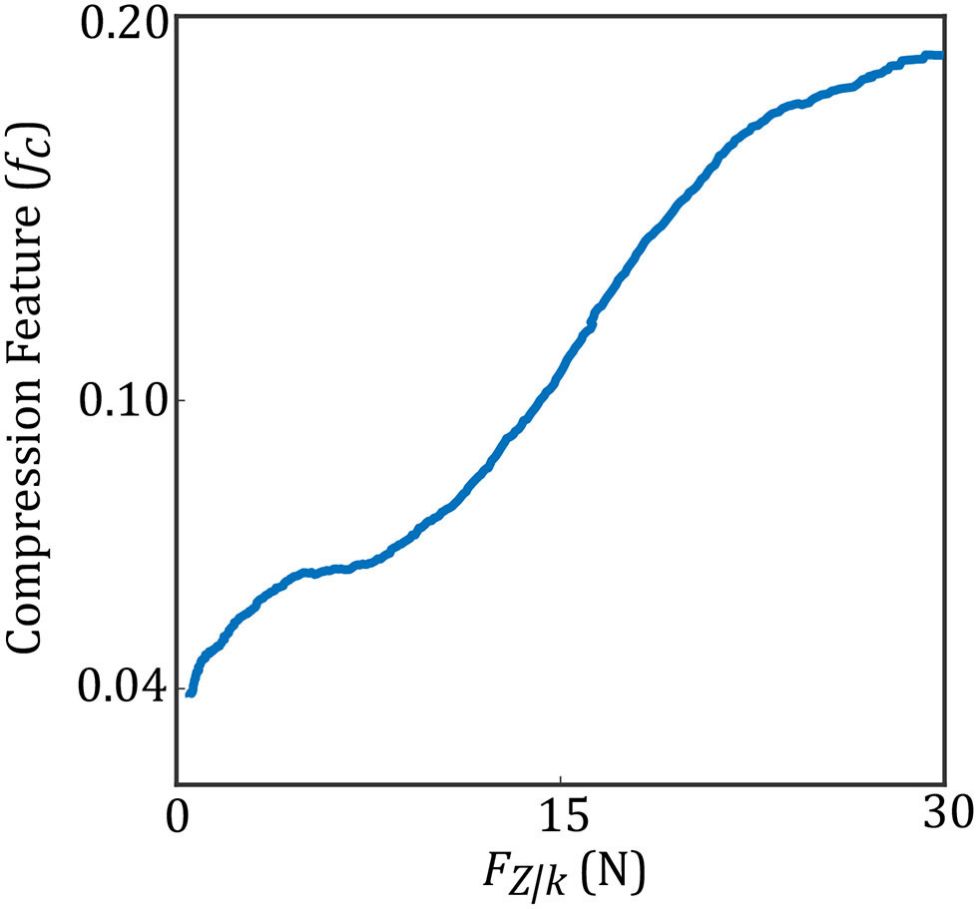
Compression feature with respect to measured force.

In (3), *U* and *L* are the maximum and minimum location of the pixels having intensity higher than *t*_*comp*_. We define *f*_*c*_ as the compression feature in (3). *M* is the height of the image along the y direction. Figure 3 shows two images with different levels of compression. Figure 3A is the US image with a high level of compression, and Figure 3B is the US image with a low level of compression. We have also shown a variation of *f*_*c*_ with respect to measured force in the z direction of the force sensor frame *F*_*Z*|*k*_ (this is aligned with the y direction in image frame) in Figure 4.

#### 3.1.3. Noise

As we mentioned earlier, the US image is very noisy. The noise comes from the manner in which US captures an image. This noise feature is also very important for the quality assessment of US images. As a first step, we use a Wiener filter for removing speckle noise from the US image. The calculation of the Wiener filter is based on Lim (1990). The US image's noise level can be estimated by the mean and standard deviation of the difference image between the original image *I*_*k*_ and the filtered image *I*_*k, f*_. Equations (5) to (8) show the mathematical explanation of using a Wiener filter to remove noise from the US image and calculate the noise feature.

(4) $$\begin{array}{r} \mu =\frac{\sum_{p_{x}\in \eta }\sum_{p_{y}\in \eta }I_{k}\left(p_{x},p_{y}\right)}{P\times Q} \end{array}$$

(5) $$\begin{array}{r} \sigma^{2}=\frac{\sum_{p_{x}\in \eta }\sum_{p_{y}\in \eta }I_{k}{\left(p_{x},p_{y}\right)}^{2}}{P\times Q}-\mu^{2} \end{array}$$

(6) $$\begin{array}{r} I_{k,f}\left(p_{x},p_{y}\right)=\mu +\frac{\sigma^{2}-\nu^{2}}{\sigma^{2}}\left(I_{k}\left(p_{x},p_{y}\right)-\mu \right) \end{array}$$

(7) $$\begin{array}{r} I_{n}=I_{k}-I_{k,f} \end{array}$$

(8) $$\begin{array}{r} f_{n}=\bar{I_{n}}+\sigma_{n} \end{array}$$

**Figure 5:**
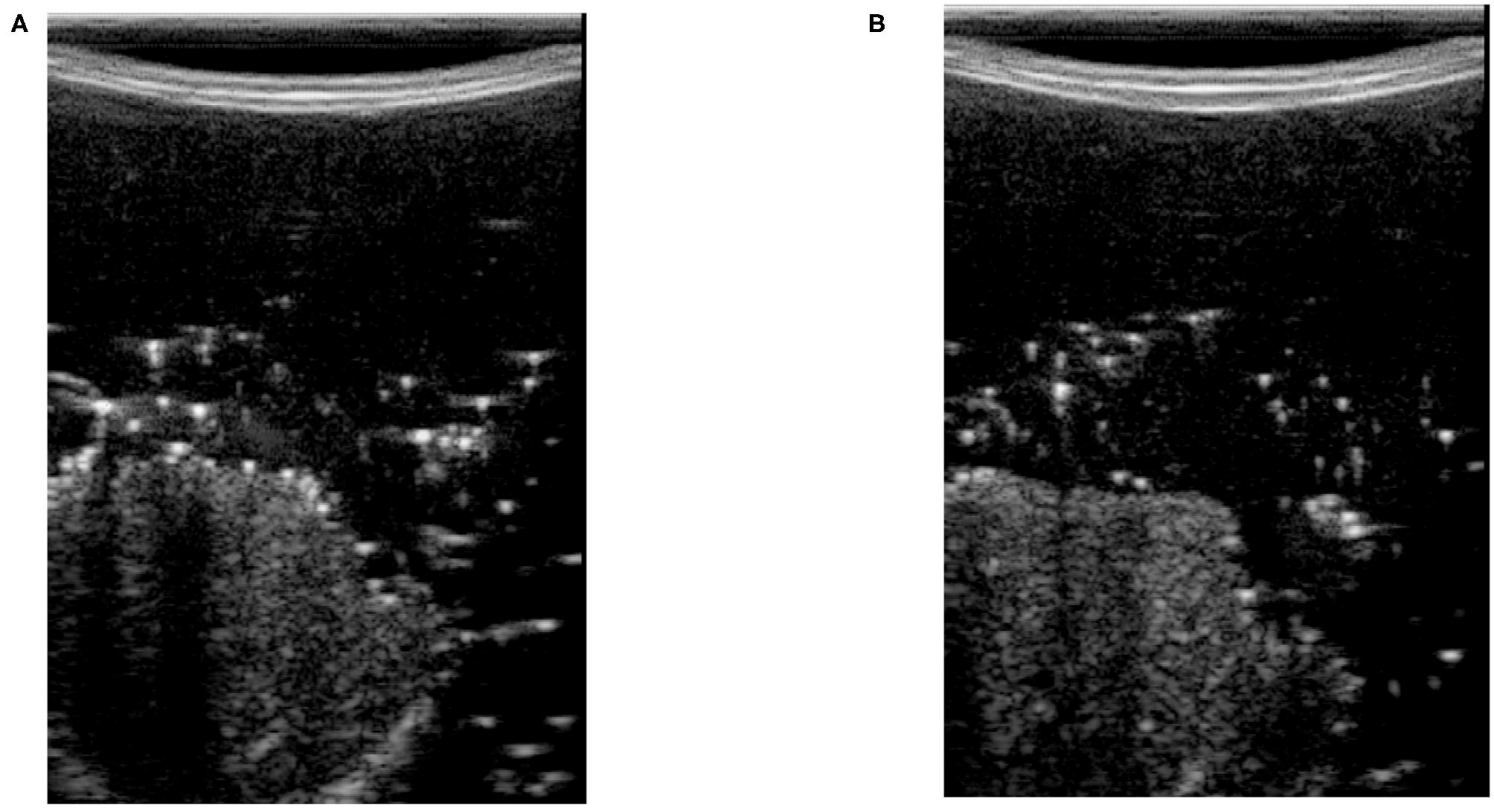
Ultrasound images with high and low levels of noise. **(A)** Ultrasound image with a high levels of noise. **(B)** Ultrasound image with a low levels of noise.

**Figure 6:**
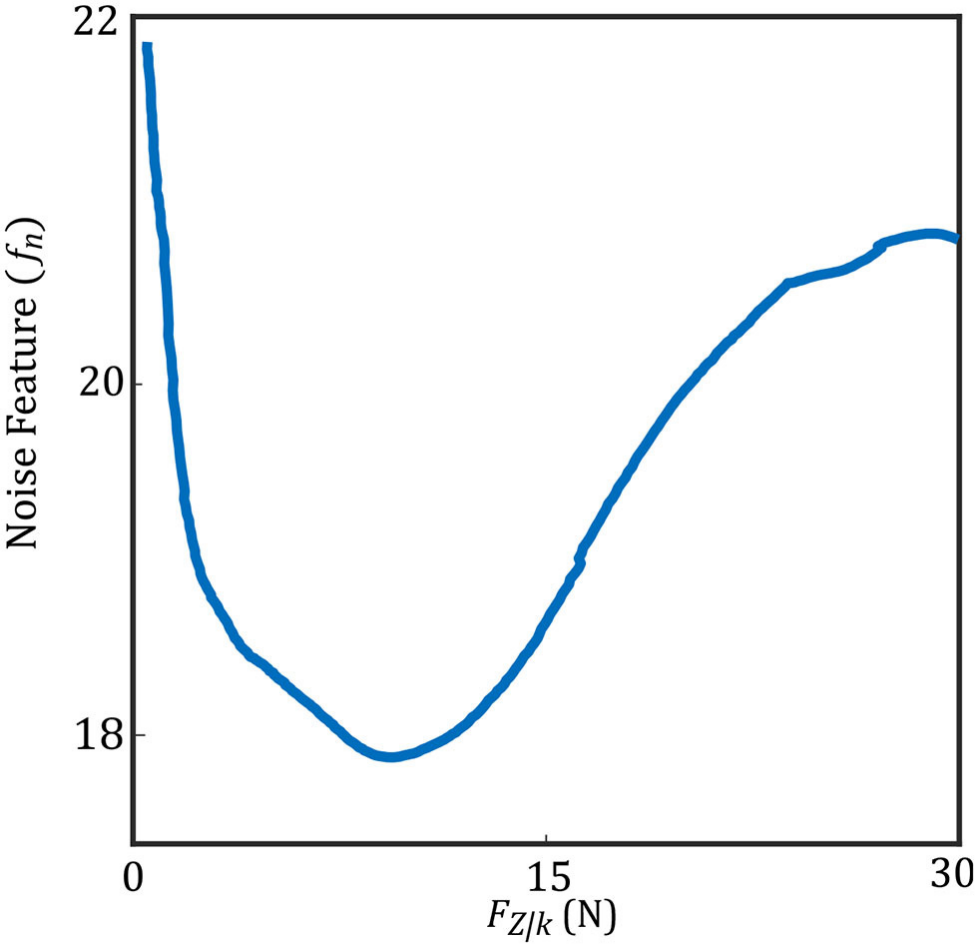
Noise feature with respect to measured force.

Here, η is the neighborhood with the size of *P* × *Q* around each pixel of the noisy image and *I*_*k*_(*p*_*x*_, *p*_*y*_) is the intensity of each pixel in the noisy US image. μ is the average of pixel intensity in the original US image, and σ^2^ is the corresponding variance value in (6). *I*_*k, f*_(*p*_*x*_, *p*_*y*_) is the intensity of the US image after removing the noise using Wiener filter and ν^2^ is the noise variance in the image in (7). Equation (8) finds the difference between US image *I*_*k*_ and filtered image *I*_*k, f*_ to find the US image's noise. In (8), $\bar{I_{n}}$ is the average of noise in the image and σ_*n*_ is the corresponding standard deviation value. Figure 5 shows two images with high level (Figure 5A) and low level (Figure 5B) of noise. We have also shown in Figure 6, the variation of the noise feature *f*_*n*_ in the US image with respect to measured force *F*_*Z*|*k*_.

### 3.2. Support Vector Machine (SVM)

The compression and noise features mentioned above will be used as an input to the SVM classifier (e.g, taking the output of the image feature calculation, Equations (3) and (8), for *I*_*k*_ we then calculate the SVM score) and the correlation feature works as a gate. SVM classifier tries to find a line that separates two classes based on the features in feature space. SVM finds this line by optimizing a cost function based on the margin between two classes in feature space. There may be a need to increase the features' dimension to find this line in a higher dimensional space.

We tested the SVM using cross-validation. We used two different tissue phantoms to train and test the SVM, meaning we trained the SVM using one of the phantoms and tested it on the other phantom. The phantoms were biological porcine and bovine tissue. We trained the SVM using bovine phantom, and the trained SVM was tested on porcine tissue and vice versa. We will use the output of the SVM for robotic control.

**Figure 7:**
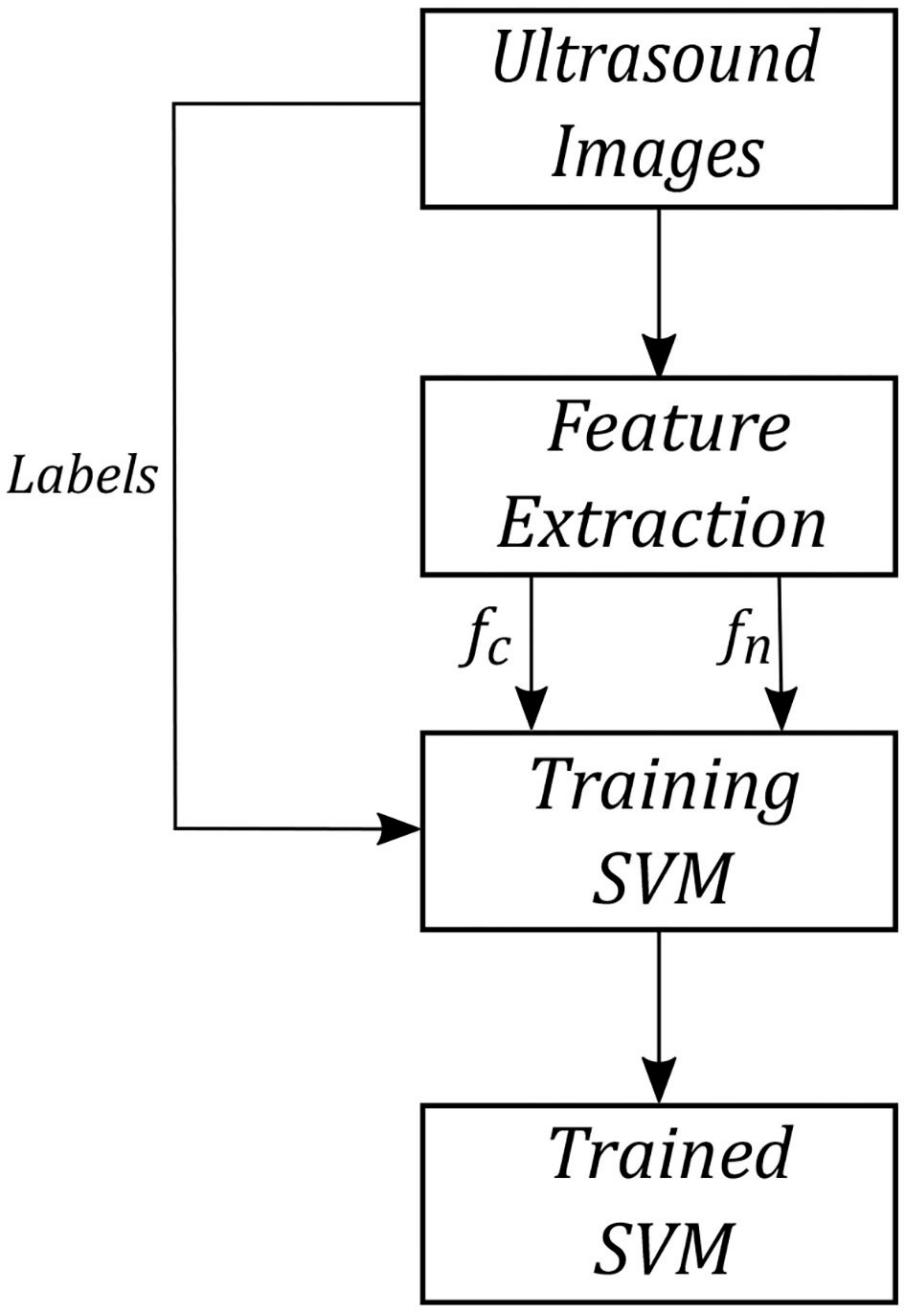
SVM training procedure.

We created an image database for training and testing the SVM. To create a database, we used a robot arm to scan bovine and porcine tissue phantoms by scanning multiple points on these tissues automatically by increasing force values at each point. The scanning procedure started from one side of the tissue and continued by dividing them to many points and increasing the US scanning force applied to the tissue from 1 to 20 *N* with an increment of 0.25 *N*. The force increment was based on force control feedback in the robotic arm by increasing the tissue indentation until the force value reached the desired force. This procedure was just used for creating a bovine and porcine image database. The images captured at each point on the tissue and the forces' value were saved using a computer. A trained non-medical user then manually classified all images and a subset of 1,000 images selected with 500 high-quality images and 500 low-quality images from the tissue phantoms' US images for different force values. The images were classified subjectively by the user, and the images were determined to be high quality if there is sufficient contact between tissue and the probe and tissue is visible without significant deformation within the US image. The variation of the pixel intensity in the frame with respect the background was also been considered for image classification. The SVM was trained using 800 images with equal probability weighting in each of the two classes. The trained SVM was tested on the remaining 200 images. After training, the SVM has reached an accuracy (a ratio of the number of correct labels to all labels) of 96% on our test database. Figure 7 shows the procedure of training SVM using biological porcine and bovine tissue.

**Figure 8:**
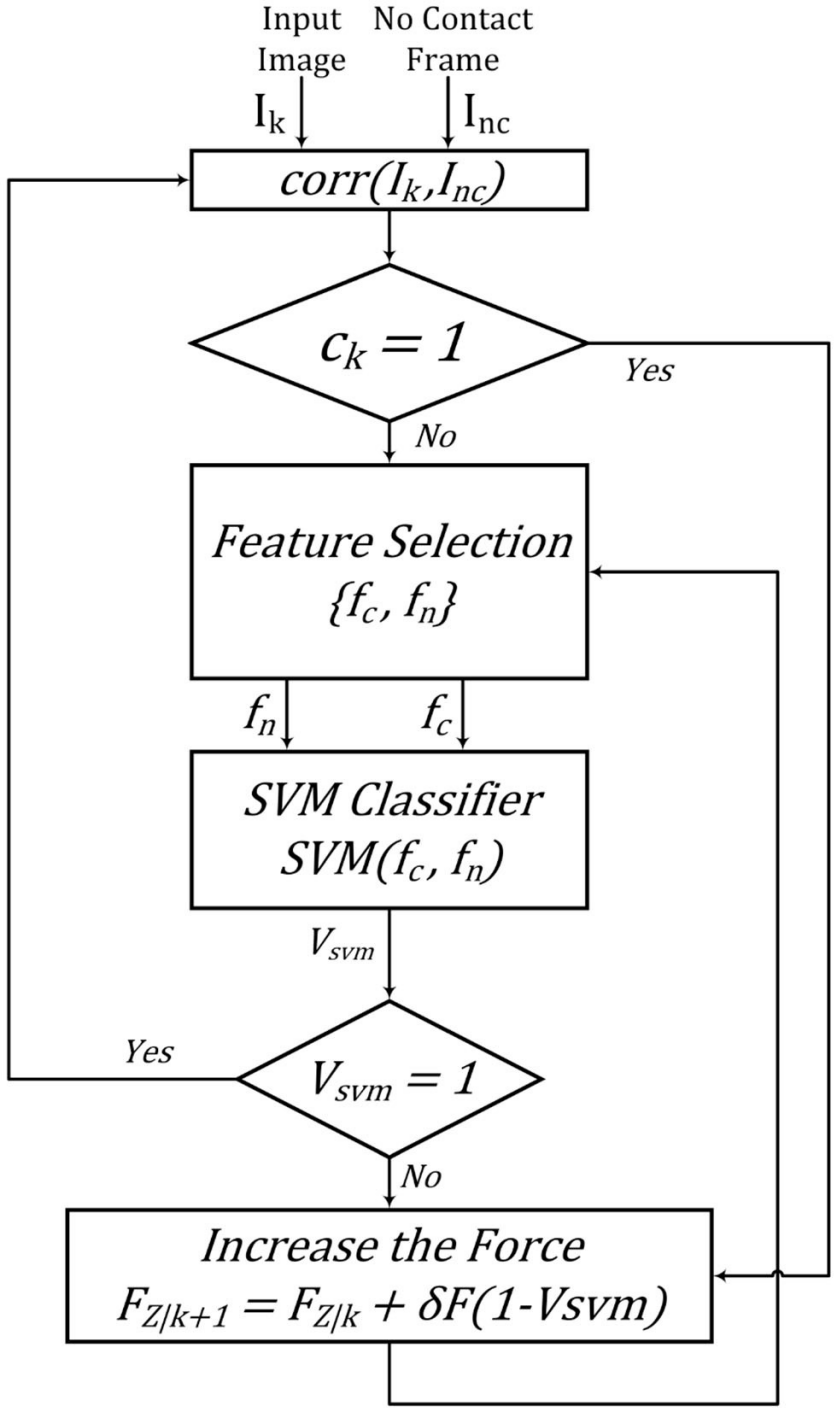
Quality assessment algorithm.

The rule for updating the force's value based on the output of the image quality assessment algorithm is shown in Equations (10) and (10). We have also shown a block diagram of the quality assessment algorithm in Figure 8.

(9) $$\begin{array}{r} V_{svm}=SVM\left(f_{c},f_{n}\right);V_{svm}\in \left\{0,1\right\} \end{array}$$

(10) $$\begin{array}{r} F_{Z|k+1}=F_{Z|k}+\delta F\left(1-V_{svm}\right) \end{array}$$

## 4. Robot Admittance Control

Our admittance controller in the x-y-z direction keeps the robot in the original x-y position and updates the z position based on the image quality assessment algorithm, as mentioned earlier. We transform the force sensor data into the base frame of the robot. Figure 1 shows the robot coordinate system during the experiments.

**Figure 9:**
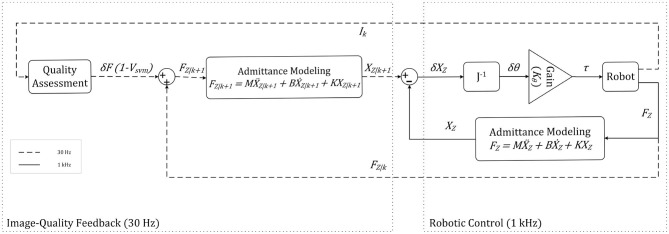
Robot control for the z axis.

We use the output of the quality assessment algorithm in the loop controlling the force applied by the US probe to tissue. Figure 9 shows the control loop for the z-axis used during the experiments. The admittance model calculates desired position of the robot based on the input force. *K*_θ_ is the gain for calculating how much torque should be applied at joints. The control loop works on two different frequencies. Dash lines in Figure 9 represent image-quality feedback working on 30 *Hz*, and the solid lines represent robotic control working on 1 *kHz*. We reduced the sampling time of robotic control to 30 *Hz* to avoid discrepancies during our experiment.

The value of the force applied to the tissue in the z-direction is fed to the admittance controller. The transfer function describes the admittance model in (11). Where *X*_*k*_(*s*) is the desired Cartesian position in the robot base frame, and *F*_*k*_(*s*) is the force applied to the end effector in the robot base frame in the z-direction. **M** is the virtual mass matrix specified for the system. **B** and **K** represent specified damping and spring matrices, respectively. The matrices **M**, **B**, and **K** are shown in section 5. The admittance model in the feedforward finds the desired position for the system, while the feedback impedance model calculates the robot's current position. We multiply the error by inverse jacobian *J*^−1^ and *K*_θ_ to find the error in joint space, and torque should be applied at joints.

(11) $$\begin{array}{r} H\left(s\right)=\frac{X_{k}\left(s\right)}{F_{k}\left(s\right)}=\frac{1}{\text{M}s^{2}+\text{B}s+\text{K}} \end{array}$$

For the experimental setup and results, which will be covered in (5), We chose the values of **M**, **B**, and **K** for the parameters of the admittance model, as shown in the following matrices. The matrix of **K** has only one non-zero parameter (in the z direction) that controls the US force applied to the tissue. The values of **M** and **K** are based on Piwowarczyk et al. (2020), and they were chosen empirically as a trade-off between sluggishness and control of the system. We calculated the value for **B** to have a critically damped response in the z direction. The threshold values in our quality assessment algorithm were found empirically based on the SVM response in our US image database, these values are *t*_*corr*_ = 0.7 and *t*_*comp*_ = 20.

(12) $$\begin{array}{r} \text{M}=\left[\begin{array}{ccc} 5.625 & 0 & 0\\ 0 & 5.625 & 0\\ 0 & 0 & 5.625 \end{array}\right]kg \end{array}$$

(13) $$\begin{array}{r} \text{B}=\left[\begin{array}{ccc} 33.54 & 0 & 0\\ 0 & 33.54 & 0\\ 0 & 0 & 33.54 \end{array}\right]\frac{N\cdot sec}{m} \end{array}$$

(14) $$\begin{array}{r} \text{K}=\left[\begin{array}{ccc} 0 & 0 & 0\\ 0 & 0 & 0\\ 0 & 0 & 50 \end{array}\right]\frac{N}{m} \end{array}$$

## 5. Experimental Setup and Results

In this study, an Axia80-M20 force-torque sensor (ATI Industrial Automation, Apex, NC, USA) was mounted on a Panda robotic arm (Franka Emika GmbH, Munich, Germany), which holds US probe (see Figure 1). We have used US machine for capturing images with an Epiphan DVI2USB3.0 (Epiphan Systems Inc, California, USA) for sending the image to the computer. The US machine used for the experiment was an Ultrasonix Touch with a 4DL14-5/38 Linear 4D transducer (Ultrasonix Corp, Richmond, BC, Canada). For this experiment, we only use the 2D functionality of the US probe. We used a tissue phantom made of plastisol as an artificial tissue for our experiment. The setup is shown in Figure 1.

The admittance controller was programmed and implemented in MATLAB 2019a (The Mathworks Inc., Natwick, MA, USA) and ran using Simulink on a PC running Ubuntu 16.04 LTS. The PC has an Intel Core i5-8400 running at 4.00 GHz. The communication between robot and computer was done over UDP, and the Epiphan was connected to the computer using a USB port.

To evaluate the image quality controller algorithm, we selected six spots on the surface of the plastisol tissue and ran the proposed method on those six locations. We then manually classified the acquired images and found the values of Structural Similarity Index Metric (SSIM) and Peak Signal to Noise Ratio (PSNR) between the output of our quality assessment algorithm and our manual subjective results. The calculation of SSIM is based on Wang et al. (2004). These values are reported in Table 1.

**Table 1:** Similarity metrics' value between quality assessment algorithm and subjective classification.

**Location**	**SSIM**	**PSNR**
First position	0.87	26.85
Second position	0.76	20.60
Third position	0.84	24.30
Fourth position	0.88	28.16
Fifth position	0.86	24.53
Sixth position	0.82	22.54

**Figure 10:**
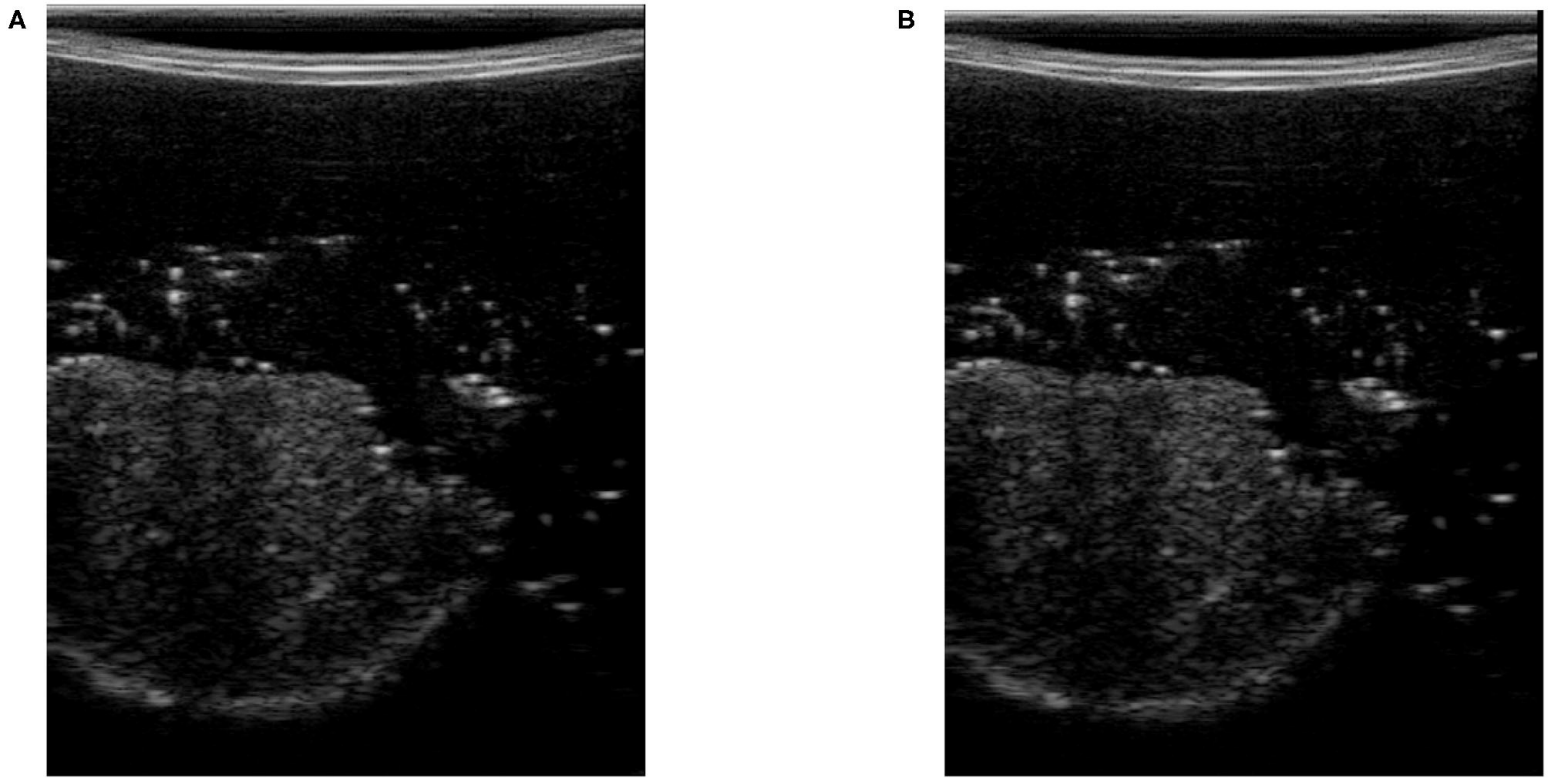
Output of quality assessment algorithm and human subjective classification. **(A)** Quality assessment output. **(B)** Subjective result.

The experiments are designed to test the feasibility of incorporating our quality assessment algorithm into the control loop. The robot increases the force applied to the tissue by going down in the z-axis using an admittance controller. Figure 10 shows the output of the quality assessment algorithm and the subjective result by the human operator. Figure 10A is the output of the quality assessment algorithm in one specific position and Figure 10B is the output of the manual classification of the image in that specific position. This will show that our proposed method provides US images of high quality similar to those taken by a sonographer.

The values reported in Table 1 show the US image captured using our proposed image quality assessment method is similar to the result of manual classification. The similarity between the values of SSIM and PSNR in all six positions proves the generality of the proposed quality assessment method. Being as PSNR only compare the values of intensities without analyzing general features of the image like the shape of the organ inside the tissue. The SSIM finds the similarities between two images based on structural analysis. The values of SSIM are high for our experiment, which proves our algorithm performs very close to a human operator.

**Figure 11:**
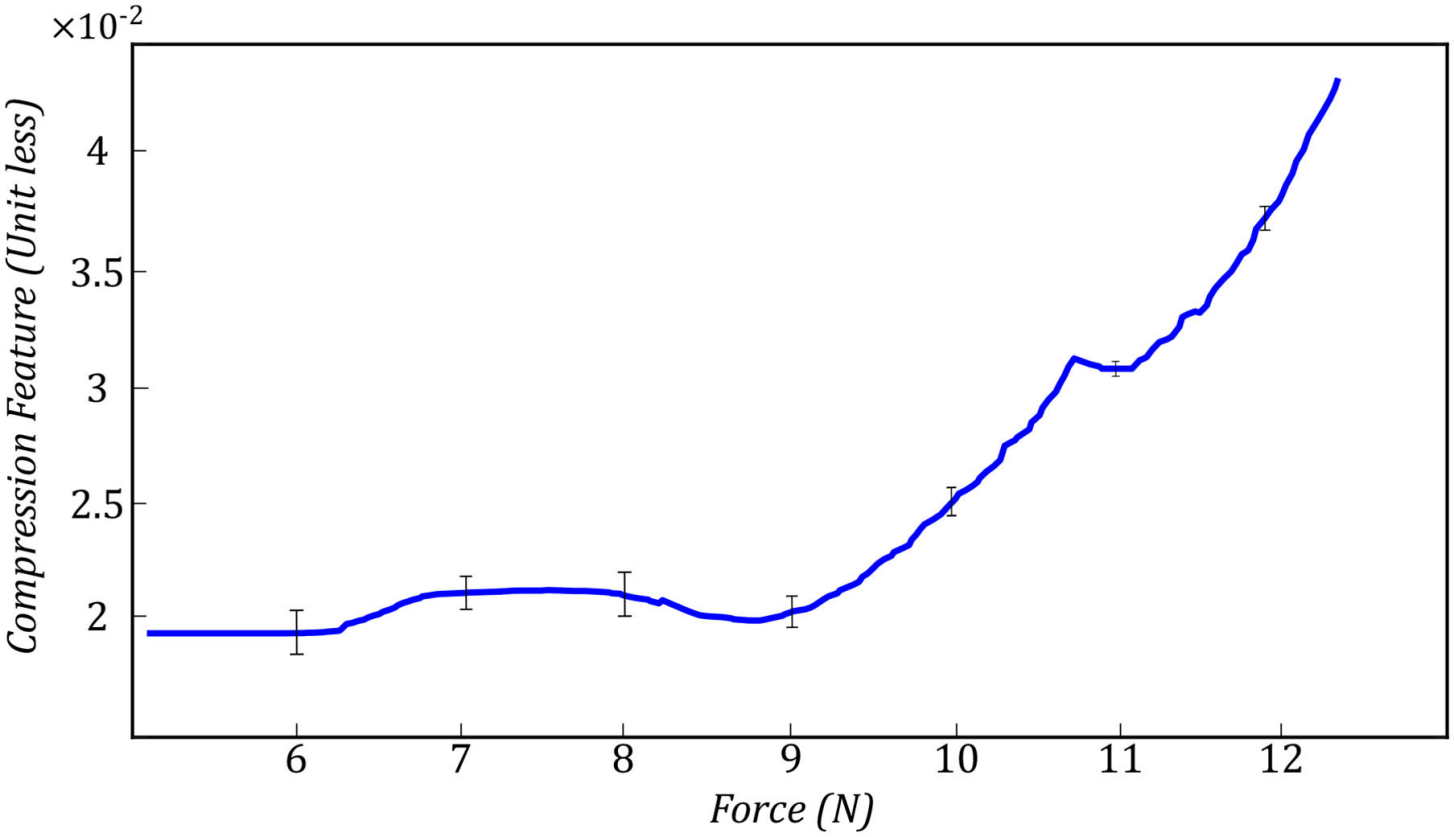
Variation of compression feature during the test experiment in all six spots on the surface of the tissue.

**Figure 12:**
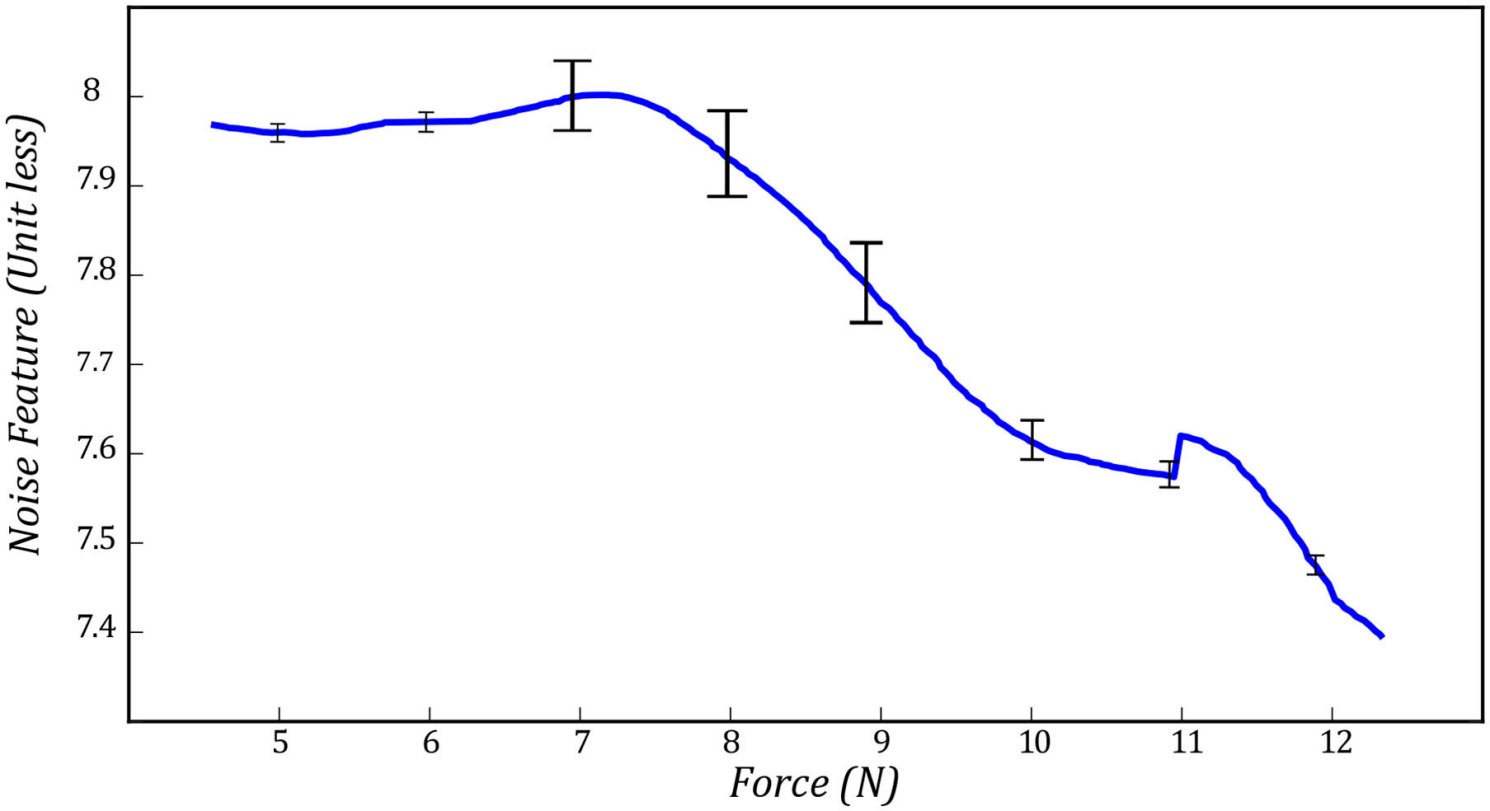
Variation of noise feature during the test experiment in all six spots on the surface of the tissue.

**Figure 13:**
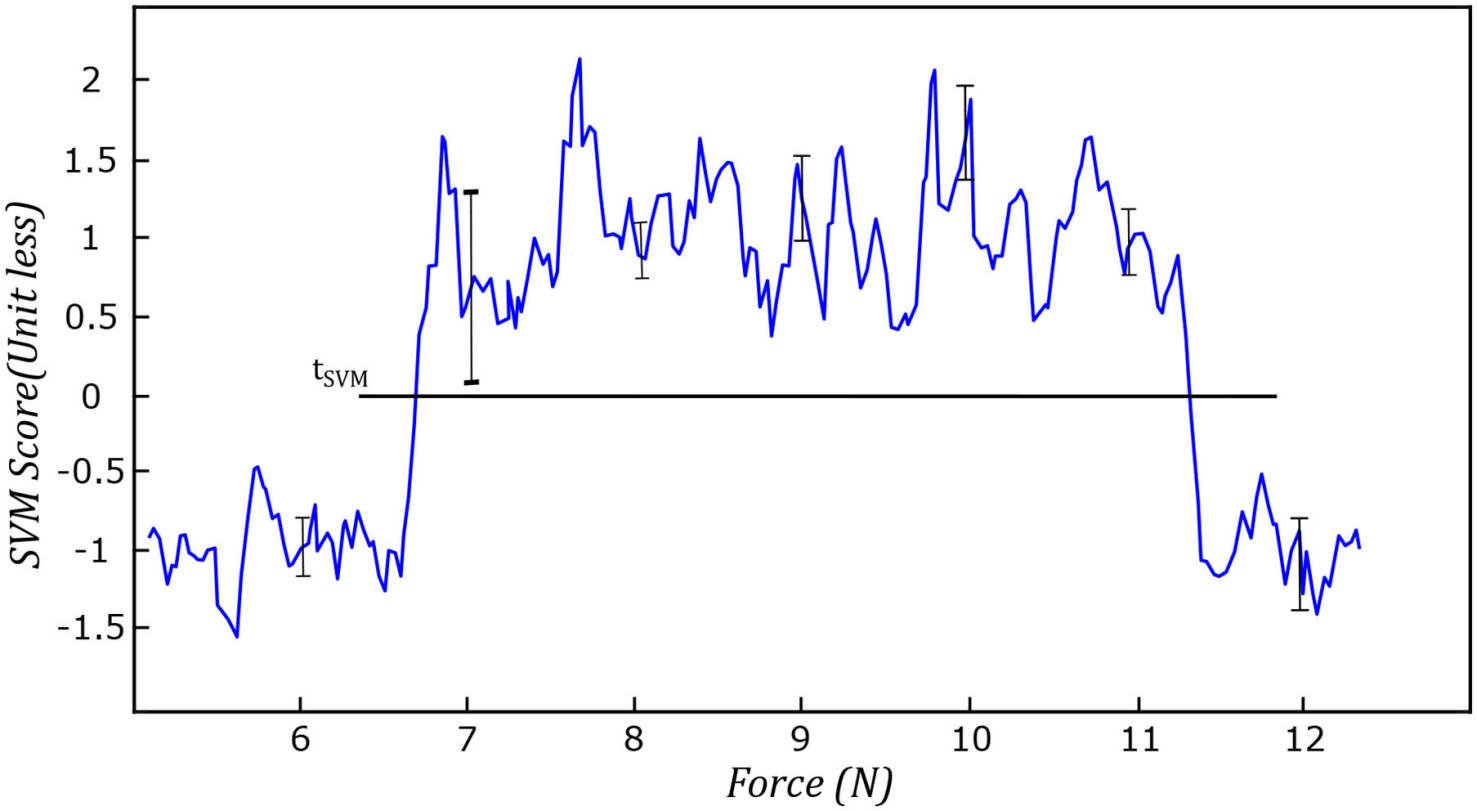
Variation of SVM during the test experiment in all six spots on the surface of the tissue.

We evaluated the performance of the proposed method experimentally by recording the values of each feature and the output of SVM by controlling the force applied to the tissue. Figure 11 shows the average value of compression value with respect to the force applied to the tissue during the test experiment. The values reported in this figure, are the average compression feature values in six different spots on the surface of the tissue. The bar in each force value represents the variation of the compression feature at the corresponding force value at all six locations on the tissue. We also reported the same variation for noise feature in Figure 12. Figure 13 shows the variation of SVM output during scanning of the tissue by increasing the force applied to it. The threshold value of *t*_*SVM*_ divides the graph to two separate classes in which the top part is associated with class of high-quality images and the bottom part is related to the low-quality images. These graphs prove the generality of our proposed method in different situations as the variation of each feature across the different levels of force was within the limited range in all six locations on the tissue.

The experiments conducted in this section shows us that the level of force applied to the tissue using the quality assessment algorithm is within a reasonable range, based on the results shown in Figures 11–13. The general trend and variation of these features during scanning are consistent with respect to the applied force, which proves the generality of the proposed method. Figure 10 and Table 1 show us that the output of the quality assessment algorithm is very close to the desire of the sonographer that all the values reported in Table 1 are within a reasonable range and the image acquired using image quality assessment algorithm and the subjective result are very close to each other in Figure 10.

## 6. Conclusion

This paper has presented US image quality assessment algorithm used for robotic control of US scanning. Our proposed quality assessment algorithm uses feature extraction and a SVM classifier to assess the acquired images' quality. The algorithm estimates the US image's quality based on correlation, compression, and noise features. These features are input into a SVM classifier to determine an image is of high quality or low quality. The algorithm was used as a part of the real-time control loop in the robotic US image scanning system. The user is able to put the US probe at a specific location on the tissue, and the algorithm will modulate the US scanning force applied to the tissue. An admittance controller was used internally to modulate the force. We evaluated the performance of the proposed system using different quality assessment metrics, showing close agreement between manual subjective assessment of the captured US image quality and the quality estimation from our algorithm.

This system is designed to enable isolation between patients and sonographers during the COVID-19 pandemic. In the future, we can control the US probe's orientation in an autonomous manner to enable six degrees of freedom of the US probe during scanning. We can also incorporate the quality assessment algorithm into a teleoperation system to enable remote control of a US scanning robot. Here, the user can remotely move the robot to the desired location, with the algorithm appropriately adjusting the US scanning force automatically.

## Data Availability Statement

The raw data supporting the conclusions of this article will be made available by the authors, without undue reservation.

## Author Contributions

This paper has been prepared by MA under the supervision of MT and JC. TM, RS, SH, and NU helped the research group by giving advisory comments. All authors contributed to the article and approved the submitted version.

## Conflict of Interest

The authors declare that the research was conducted in the absence of any commercial or financial relationships that could be construed as a potential conflict of interest.
